# Amplicon sequence data from the gut microbiota of broiler chickens fed black soldier fly (*Hermetia illucens*) larvae-based diets

**DOI:** 10.1128/mra.01230-25

**Published:** 2026-01-16

**Authors:** Deborah O. Ibiwoye, Samuel O. Dahunsi

**Affiliations:** 1 Microbiology Programme, College of Agriculture, Engineering, and Science, Bowen University Iwo 683778, Iwo, Osun, Nigeria; 2 The Radcliffe Institute for Advanced Study, Harvard University 53785, Cambridge, Massachusetts, USA; Indiana University, Bloomington, Bloomington, Indiana, USA

**Keywords:** gut microbiota, black soldier fly, broiler chicken, 16S rRNA, amplicon sequencing

## Abstract

This manuscript describes 16S Ribosomal rRNA gene amplicon sequences from gut samples of broiler chickens fed diets containing 0%, 25%, 50%, and 100% black soldier fly larvae. The data set includes raw reads, amplicon sequence variants, and taxonomic assignments supporting microbiome analysis under insect-based feeding regimes.

## ANNOUNCEMENT

According to current global population trends, there will be around 9.2 billion people on the planet by 2050, which will raise the need for protein, particularly poultry meat (1). Poultry, specifically broilers, can close the gap between the supply and demand for protein (2). Broiler chickens efficiently convert feed into 2.5–3.5 kg of meat within 6–8 weeks, highlighting their importance (3). Demand for sustainable poultry feed ingredients has intensified interest in black soldier fly larvae (BSFL) as an alternative protein source (4, 5). To characterize microbial responses to BSFL diets, 16S rRNA gene amplicon data were generated from broiler gut samples across four dietary treatments: T1 (0% BSFL), T2 (25%), T3 (50%), and T4 (100%).

DNA was extracted using the ZymoBIOMICS DNA Miniprep Kit (Zymo Research) following the manufacturer’s standard protocol, with no deviations.

The full V1–V9 region of the bacterial 16S rRNA gene was amplified using primers:

- **27F**: AGAGTTTGATCMTGGCTCAG.
- **1492R**: TACGGYTACCTTGTTACGACTT.

M13-tailed adapters were used for PacBio barcoding.

PCR conditions were initial denaturation at 95°C for 3 min; 30 cycles of 95°C for 30 s, 55°C for 30 s, and 72°C for 90 s; final extension at 72°C for 5 min. Each reaction contained 10–20 ng template DNA and Q5 High-Fidelity polymerase (New England Biolabs). Amplicons were quantified using Qubit dsDNA assays, normalized equimolarly, pooled, and purified with AMPure PB beads.

SMRTbell library preparation and sequencing were performed on the PacBio Sequel IIe platform according to the manufacturer’s protocol.

Read processing was performed in QIIME 2 (v2024.2) using the DADA2 plugin with the following parameters:

- **Minimum read quality (Phred):** Q20.
- **Maximum expected errors:** 2.
- **Truncation:** No truncation was applied due to the high-quality PacBio CCS reads.
- **Chimera removal:** “consensus” method.
- **ASV inference:** DADA2’s single-nucleotide resolution model was used, allowing differentiation of sequence variants differing by as little as one nucleotide (i.e., ASVs represent exact biological sequence variants rather than 97% clusters).

Although full-length 16S rRNA gene sequences (V1–V9) were generated, taxonomic assignment in QIIME 2 was conducted using a V3–V4–trimmed SILVA 138 classifier. This approach was selected because QIIME 2’s naïve Bayes classifiers require region-specific training for optimal accuracy, and the V3–V4 region currently offers the highest taxonomic resolution and best-supported reference models for poultry microbiome data sets. Full-length classifiers remain less reliable for certain taxa due to uneven alignment quality across hypervariable regions.

A total of four representative pooled samples were sequenced: T1: 127,847; T2: 52,000; T3: 112,358; and T4: 104,744.

Across samples, reads resolved into unique ASVs representing major bacterial phyla typical of poultry gut microbiota, including Firmicutes, Bacteroidota, and Proteobacteria. The data set provides baseline taxonomic profiles supporting future comparative analyses of BSFL diets.

## Data Availability

Sequence data are accessible in the GenBank of the National Center for Biotechnology Information (NCBI) database under accession numbers: SRX30931451, SRX30931452, SRX30931453, and SRX30931454 (https://www.ncbi.nlm.nih.gov/bioproject/PRJNA1339025).
